# Congenital epicardial coronary artery to bilateral internal mammary artery fistulae

**DOI:** 10.1259/bjrcr.20160014

**Published:** 2016-11-02

**Authors:** Arjun Kumar Ghosh, Sze Mun Mak, Yousif Ahmad, Iqbal Malik, Petros Nihoyannopoulos, Deepa Gopalan

**Affiliations:** ^1^Cardiology Department, Barts Heart Centre, St Bartholomew’s Hospital, Barts Health NHS Trust, London, UK; ^2^Radiology Department, Hammersmith Hospital, Imperial College Healthcare NHS Trust, London, UK; ^3^Cardiology Department, Hammersmith Hospital, Imperial College Healthcare NHS Trust, London, UK

## Abstract

A 39-year-old male ex-smoker gave a history of exertional chest pain ever since he suffered a respiratory tract infection. Clinical examination, electrocardiogram and echocardiography were normal and he was referred for a cardiac CT scan to assess coronary artery calcification and patency. The scan demonstrated incidental fistulae between the right internal mammary artery and the right coronary artery, and the left internal mammary artery and the left anterior descending artery.

## Clinical presentation

**Figure 1.**
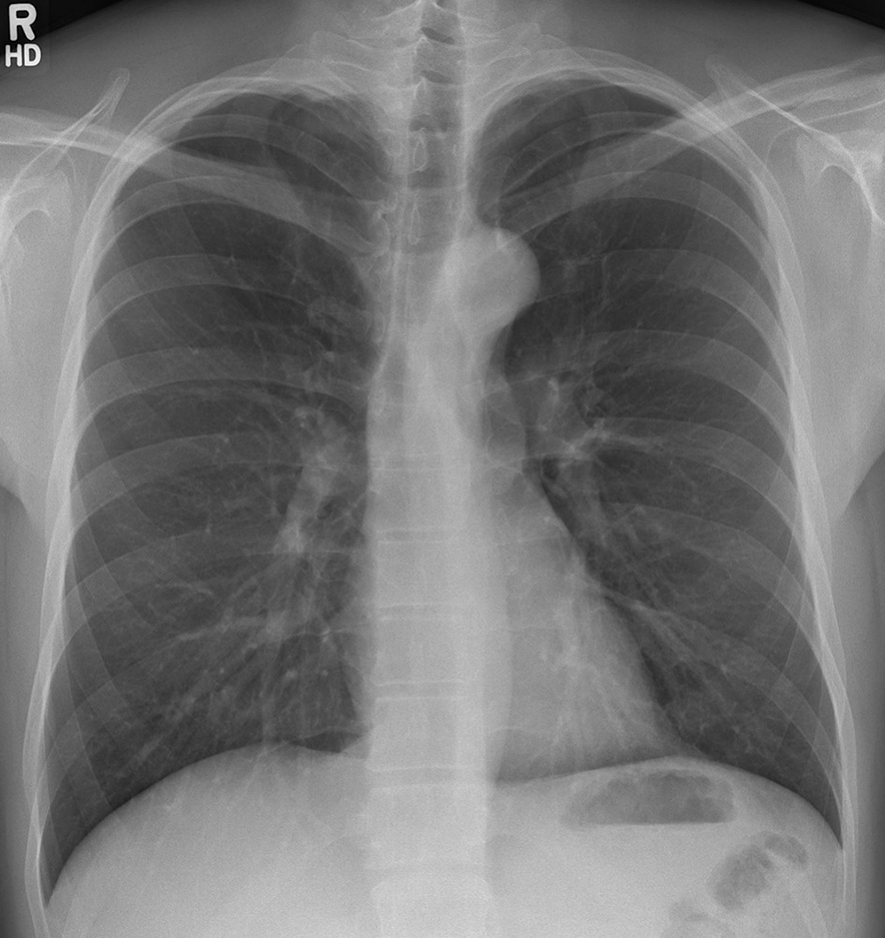
Chest radiograph (posteroanterior projection).

**Figure 2.**
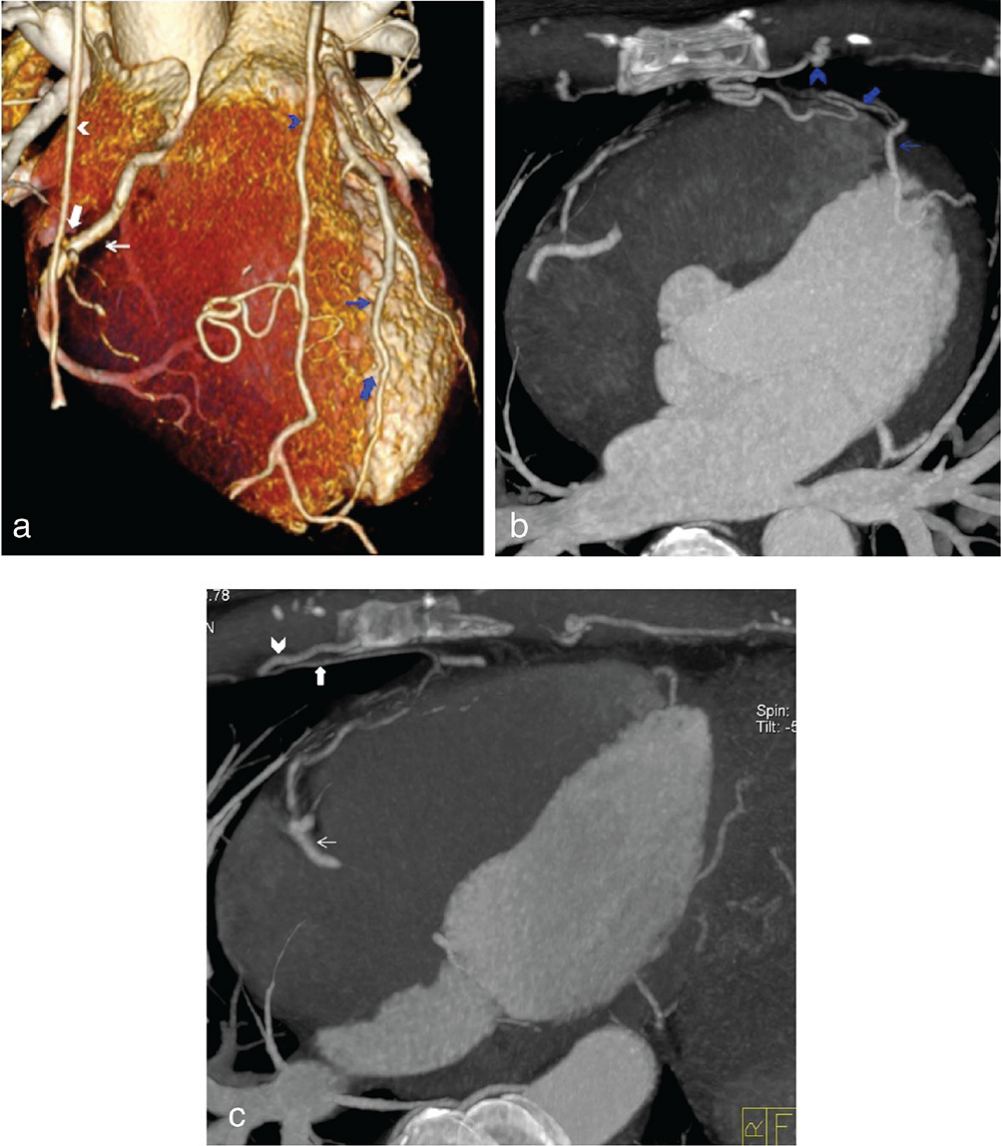
(a) Volume-rendered cardiac CT image demonstrates the fistulous communication (white and blue block arrows) between the right internal mammary artery (white chevron) and mid-right coronary artery (thin white arrow), and left internal mammary artery (thin blue chevron) and mid left anterior descending artery (thin blue arrow). (b, c) CT axial curved maximum intensity projections demonstrating the fistulous communication (white and blue block arrows) between the right internal mammary artery (white chevron) and mid-right coronary artery (thin white arrow), and left internal mammary artery (thin blue chevron) and mid left anterior descending artery (thin blue arrow).

A 39-year-old male ex-smoker gave a history of exertional chest pain ever since he suffered a respiratory tract infection. Similar symptoms after a previous chest infection a few years ago had cleared spontaneously. He was reviewed in the respiratory clinic and found to have no lung disease and then referred to cardiology. Clinical examination, electrocardiogram, echocardiography and chest radiograph (Figure 1) were normal and he was referred for a cardiac CT scan to assess coronary artery calcification and patency (Figure 2a–c).

CT scan with volume-rendered and axial curved maximum intensity projection techniques elegantly demonstrated the fistulous communication (white and blue block arrows) between the right internal mammary artery (white chevron) and mid-right coronary artery (thin white arrow) and left internal mammary artery (thin blue chevron) and mid left anterior descending artery (thin blue arrow) (Figure 2). The patient’s epicardial coronary arteries were of normal origin and calibre and free from atheroma with right dominant circulation. The calcium score was zero.

**Figure 3.**
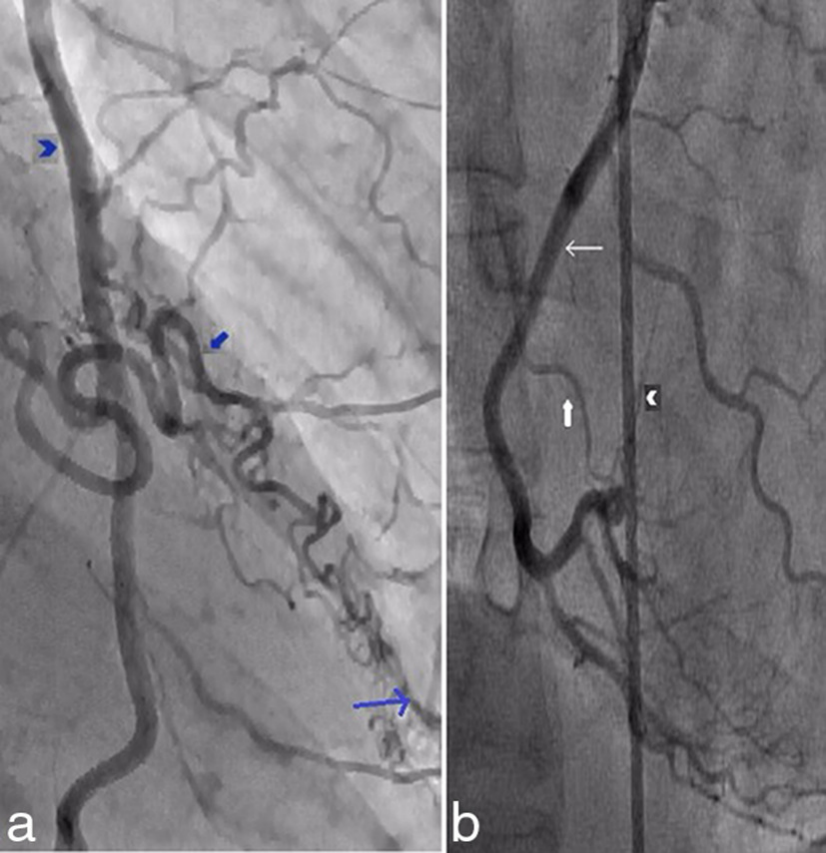
(a) Coronary angiogram image demonstrating the fistulous communication (blue block arrow) between the left internal mammary artery (blue chevron) and mid-left anterior descending artery (thin blue arrow). (b) Coronary angiogram image demonstrating the fistulous communication (white block arrow) between the right internal mammary artery (white chevron) and mid-right coronary artery (thin white arrow).

A subsequent stress cardiac MR scan demonstrated normal cardiac function and no inducible perfusion defects (Supplementary Video A). Coronary angiography confirmed patent coronary arteries and the presence of the fistulae (Figure 3a,b and Supplementary Videos B and C).

## Discussion

This is an extremely rare phenomenon and the first description in the literature of congenital epicardial coronary artery to bilateral internal mammary artery fistulae. Congenital fistulae of the vasculature are rare and usually involve communications between coronary arteries and chambers of the heart (coronary–cameral fistula) or pulmonary circulation (coronary arteriovenous fistula).^1^ Fistulae involving the mammary arteries can be secondary to intervention most commonly post coronary bypass surgery where they connect to the pulmonary arteries.^2^

The patient’s symptoms resolved spontaneously and it was felt that his fistulae were not of functional significance. He was reassured and discharged from regular review.

## Learning points

1. Congenital coronary fistulae are rare and do not necessarily have functional significance. Common communications are to the cardiac chambers or pulmonary circulation.
2. Fistulae involving the mammary arteries may be congenital or secondary to intervention.

## Consent

Informed consent to publish this case (including images and data) was obtained and is held on record.
